# ResSGA-Net: A deep learning approach for enhanced brain tumor detection and accurate classification in healthcare imaging systems

**DOI:** 10.1016/j.jgeb.2026.100658

**Published:** 2026-01-15

**Authors:** Yucheng Guan, Ahmad Alshammari, Yu Wang, Jahan Zeb Gul, Azhar Imran

**Affiliations:** a Northeastern University, San Jose, USA; b Northern Border University, Department of Computer Sciences, Rafha, 91911, Kingdom of Saudi Arabia; c Shandong Research Institute of Industrial Technology, Jinan, China; d Maynooth University, Department of Electronic Engineering, Maynooth, Ireland; e Beijing University of Technology, Department of Computer Science, Beijing, 100124, China

**Keywords:** Brain tumor classification, Deep learning, MRI images, ResSGA-Net

## Abstract

Accurate and reliable brain tumor classification from magnetic resonance imaging (MRI) is a critical component of computer-aided diagnosis systems, directly impacting clinical decision-making and patient outcomes. This study presents ResSGA-Net, a hybrid deep learning framework that integrates a ResNet50 backbone with dual attention mechanisms (global and gated) and a Swin Transformer to capture both fine-grained local features and long-range contextual dependencies effectively. A fusion strategy is employed to unify convolutional, attention-refined, and transformer-enhanced representations into a robust feature space for multi-class classification. The proposed model is evaluated on two publicly available benchmark datasets, including a four-class and a three-class brain tumor classification task, using stratified cross-validation. Extensive quantitative analysis demonstrates that ResSGA-Net achieves state-of-the-art performance, with accuracies exceeding 98% on Dataset I and strong generalization on Dataset II (accuracy of 93.18% and macro-averaged AUC of 0.989). Comprehensive statistical significance testing confirms that the observed improvements are highly significant and not attributable to random chance. Ablation studies further validate the individual contributions of attention mechanisms and data augmentation strategies, demonstrating that performance gains arise from tumor-specific feature learning rather than artificial data diversity. Qualitative analyses, including confusion matrices, training dynamics, ROC curves, and confidence-based visualizations, confirm stable convergence, robust generalization, and reliable decision confidence across tumor classes. These results indicate that ResSGA-Net provides an accurate, stable, and clinically meaningful solution for automated brain tumor classification, with strong potential for integration into real-world diagnostic imaging workflows.

## 1 Introduction

Brain tumors emerge from abnormal growth of tissue in the cranial cavity which is also a major worldwide issue due to the negative impact on quality of life.^1^ Brain tumors can be categorized into benign and malignant (the malignant brain tumors tend to be more aggressive). Malignant brain tumors (gliomas, pituitary tumors, and meningiomas) tend to be more troubling than benign brain tumors.^2^ As malignant brain tumors invade other parts of the brain, timely and accurate detection is important so that treatment can start quickly and so the client’s prognosis will not deteriorate further. Brain tumor imaging includes many forms of imaging, including MRI, Computed Tomography (CT), Positron Emission Tomography (PET), and Single Photon Emission Computed Tomography (SPECT).^3^

MRI currently stands as the commonest imaging modality providing the greatest magnification and the most detailed resolution of soft brain structure, is non-invasive and without ionizing radiation risks associated with CT.^4^ However, the MRI interpretation process will always be limited as it is performed manually and is incredibly time consuming with limited funds which require specialists with radiological training and is subject to mistakes, especially when interpreting examples of tumors that only vary subtly in shape, size or texture. Therefore, the aim of developing automated diagnostic methods for identifying brain tumors and improving tumor detection that are efficient, accurate and fast is increasingly necessary.^5^

MRI employs a strong magnetic field to capture images of organs and tissues, offering crucial chemical and physical insights into the human body at the molecular level. The images produced by MRI scans are rendered as three-dimensional objects, depicting soft tissue through imaging variations such as FLAIR, T1, and T2.^6^ These variations enable radiologists to observe tumor characteristics, including shape, size, and location. However, due to the similarities in the appearance of tumor types, MRI can be ambiguous in distinguishing between benign and malignant tumors. When a brain tumor is diagnosed via MRI, the subsequent step typically involves obtaining a tissue sample through biopsy or surgery.^7^ Although these invasive methods are necessary for diagnosis, they pose risks of patient injury, human error, and delayed diagnosis. Therefore, the development of intelligent, non-invasive diagnostic systems utilizing MRI images to segment and classify tumors is imperative for modern medicine. These systems have the potential to enhance diagnostic accuracy, reduce the risk of human error, and provide rapid results, thereby facilitating early therapeutic intervention for improved patient outcomes.^8^

The concepts of artificial intelligence (AI), encompassing image processing algorithms, machine learning, and deep learning, have garnered significant attention among researchers in the field of human health.^9^ Deep learning, a subset of machine learning, is predicated on methodologies derived from artificial neural networks (ANN). Since the advent of deep learning and the development of deep neural networks, it has become possible to assist medical professionals with tasks such as automated disease detection, classification, image retrieval, and monitoring of disease progression. These applications have proven particularly pertinent in the classification of brain tumors, where deep learning models are increasingly being employed.^10^ CNNs, the most prevalent deep learning model, have demonstrated exceptional performance in achieving high-level classification results for complex problems. Owing to their superior performance compared to traditional methods, CNNs are considered one of the most effective tools for automating image processing by automatically extracting features from images.^11^ This capability enables CNNs to derive meaning from pixels without manual intervention. Despite their efficacy in training models, CNNs have limitations, including the inability to capture global contextual information and variability in the appearances of the same tumor. These limitations may lead to reduced accuracy in classification tasks, particularly with small or unbalanced datasets. Research is ongoing to enhance CNN-based models for the development of brain tumor classification models that are both accurate and capable of yielding reliable results.^12^

There are various approaches that have been suggested to improve the automated, intelligent classification of brain tumors, which can include CNNs.^13^ Some past studies required the first step to be the identification and delineation of the abnormal tumor region, which was then utilized for the classification, as a separate part of the neural network’s work. While this provided reasonable results in both speed and accuracy in the training stage, the manual preprocessing was cumbersome. As development of neural network architectures began to emerge, new models showcased encouraging results in automated classification by eliminating the manual delineation.^14^

In response to these challenges, researchers have begun exploring more complex approaches, such as attention models,TL and multi-scale feature extraction.^15^ TL and pre-trained models have shown promise in improving feature extraction from MRI scans. This is particularly beneficial in cases of limited training data, where a model is at a high risk of overfitting or undertraining.^9^ Attention models have been effective at letting models focus on features that contribute to differentiation, like edges and areas of interest, which enhance the features of the tumor and differentiate other features of the brain, that led to better classification accuracy. These developments have certainly improved the accuracy and efficiency of brain cancer classification.^16^

In recent years, the classification of brain tumors has improved rapidly through the use of transformer-based models (e.g., Swin Transformer) that take advantage of local and global features of medical datasets. These models achieved better classification accuracy, generalizability, and computational efficiency, allowing the models to handle complex tasks, such as that of brain tumor classification.^17^ Potential improvements included augmentation data and advanced pre-processing techniques (e.g., adaptive intensity normalization and multi-path CNN structures) to promote model robustness and reliability during training and development. The methodologies were used to tackle variability and noise associated with images leading to models trained on representative and rich datasets that are ready for clinical application.^18^

Attention mechanisms, especially both global and gated attention mechanisms, have also enhanced the efficacy of deep learning models for medical image analyses and in particular cases of brain tumor classification. The global attention provides a means for the model to recognize critical areas of the image while accounting for relationships over the full image, maintaining dependencies that are also important for describing the complexity of the tumor.^19^ This is especially true for brain tumor classification because of the variation in shape and size of tumors, even with various anatomical regions of the brain. Gated attention mechanisms use gates to focus on visually relevant areas of the image, thus calibrating the feature extraction ability of models to emphasize relevant features while dampening features that are less informative. These attention mechanisms contribute to improved tumor detection accuracy, especially in challenging cases characterized by similar morphology and subtle differences.^20^

A widely adopted strategy for enhancing classification accuracy involves leveraging state-of-the-art deep neural networks throughTL. These deep networks accomplish such tasks by utilizing knowledge acquired from prior training and applying it to related tasks. Although pre-trained networks demonstrate utility, they often require extended training periods due to their numerous layers and parameters. As a result, they may be impractical due to the associated hardware costs and processing times for images.^21^

An alternative promising approach involves the use of multi-path convolutional neural networks, wherein each path operates independently.^22^ Models have been proposed that enhance classification by employing distinct paths within the CNN architecture to extract features and achieve higher accuracy. Multi-path CNN architectures have surpassed traditional deep networks by offering improved accuracy and efficiency at a reduced computational cost, particularly for tumor type classification. These advancements indicate a trend toward optimizing CNN architectures, which will encompass transformer-based models such as the Swin Transformer, integrating attention mechanisms like global and gated attention, thereby enhancing brain tumor classification systems.^23^

This research endeavors to contribute to the advancement of automated classification systems for brain tumors by employing cutting-edge deep learning methodologies. By leveraging previous work with transformer models,TL, and sophisticated feature extraction techniques, the objective is to enhance the accuracy, speed, and reliability of brain tumor classification systems.^24^ The improvement of these systems is anticipated to provide clinicians with superior information, thereby facilitating more rapid and precise clinical decision-making, ultimately enhancing patient care and outcomes.

To overcome these issues, this study demonstrates ResSGA-Net, a new deep learning model used to classify brain tumors using MRI images more accurately. The main contributions offered by this research include:

• Developed ResSGA-Net, a hybrid architecture that combines ResNet50 and Swin Transformer with global and gated attention modules to extract both local and global features from brain MRI scans.
• Employed dual attention mechanisms to emphasize tumor-relevant regions and suppress background noise, improving the discriminative power of extracted features.
• Designed a robust preprocessing pipeline with image normalization and augmentation to generate high-quality training data, reduce noise, and improve generalization across MRI datasets.
• Built a lightweight, end-to-end trainable model optimized for computational efficiency, making it suitable for real-time deployment in clinical brain tumor diagnosis.

In summary, this research offers a unique, high-performing model for brain tumor classification; and importantly, the proposed combination of deep learning and traditional machine learning methods achieves an effective, robust solution in medical imaging.

## 2 Literature review

Deep learning methodologies have demonstrated exceptional efficacy in the identification and classification of brain tumors, with ongoing advancements poised to enhance treatment and patient care. In light of this progress, researchers persist in refining these algorithms to effectively detect brain cancer. Presented below is a concise overview of selected studies on brain tumor detection.

The study^25^ details a CNN-based approach for the detection of brain tumors from MRI scans. This study involved a comparative analysis of the CNN model’s performance with ResNet-50, VGG16, and Inception V3 using a data set of 3265 MRI images. Accuracy, recall, loss, and area under the curve (AUC) were used to measure the performance. The CNN model achieved an accuracy of 93.3%, a recall of 91.19%, an AUC of 98.43%, and a loss of 0.25. The CNN model outperformed the other models in every aspect and was a reliable model for early detection of brain cancer to be treated in the clinic.

The authors,^26^ developed a light weight CNN framework for the classification of brain tumors using BraTS 2020 dataset. The framework encompasses pre-processing, segmentation and classification in one pipeline. The accuracy of the model was 94.15% and achieved much faster training and less memory space than complex networks like ResNet, and Vision Transformers. Comparative analysis reveals its competitiveness, with benchmarks including support vector machines (SVM) with feature selection (97%), pre-trained CNNs (95%),TL (97.75%), and deep convolutional features (97.5%). While CNNs continue to be the gold standard, achieving accuracies exceeding 98% in certain instances, this study highlights their versatility and potential for cross-domain applications, such as ViT-GRU for spatial–temporal tasks.

The application ofTL techniques utilizing^27^ pre-trained CNN models, specifically VGG19, Inception-v3, and ResNet50, for the diagnosis of three prevalent brain tumors from MRI scans. The features extracted from these models were refined through fully connected layers to facilitate multi-class classification. Employing a benchmark dataset of MRI images, the proposed methodology achieved an average accuracy of 90%, surpassing traditional methods. The study underscores the role of deep learning andTL in enhancing diagnostic accuracy and reducing the risk of misclassifying brain tumors. The proposed^28^ Fibonacci-Net, a lightweight CNN model featuring a novel pooling strategy for the classification of brain tumors from highly imbalanced MRI datasets. The primary innovations of this model include the determination of the number of filters in convolutional layers using the Fibonacci series, the application of depth-wise separable convolutions (DWSC) to reduce model complexity, and the integration of parallel concatenation (skip connections) with an Average-2Max pooling layer to address class imbalance. When tested on three highly imbalanced MRI datasets comprising 44 tumor classes, the model achieved an accuracy of 96.2%, a precision of 97.17%, a recall of 95.9%, an F1 score of 96.5%, and a specificity of 99.9% on the 44-class dataset.

An automated methodology for predicting brain tumors,^29^ which integrates the Firefly (FF) algorithm with an interval type-II fuzzy logic system (IT2FLS) to enhance tumor delineation in complex and heterogeneous brain tissues. The FF algorithm is employed to determine the optimal positions of clusters, while the IT2FLS is utilized to provide the final clustering. This method achieved effective segmentation of brain tumors in all acquired MRI sequences from the BRATS 2017, 2018, and 2020 datasets, made a comparison with the sensitivity, specificity, and dice-overlap index (DOI), and consistently demonstrated superior performance to traditional segmentation, and particularly strong segmentation performance. This development is beneficial to oncologists because it provides a greater understanding of tumor structures, allowing for more meaningful and accurate clinical decisions.

The impact of attention mechanisms^30^ on brain tumor classification by integrating the Channel-wise Attention Mode (CWAM) with ResNet101 (national average). The ResNet101-CWAM approach utilizes attention to capture critical spatial and channel information from MRI images, thereby addressing the limitations inherent in traditional deep learning models. When compared to conventional models such as ConvNet on the same dataset, the ResNet101-CWAM method exhibits superior performance, achieving 99.83% accuracy, 99.21% recall, 99.01% precision, 99.27% F1-score, and 99.16% AUC. This study underscores the advancements in attention networks, enhancing diagnostic confidence.

A comprehensive framework for the classification of brain tumors by integrating TV-L1 image enhancement^31^ with MRI segmentation and employing feature extraction techniques such as MSLBP, QWT, and HOG. Subsequently, feature selection is conducted using the MRMR method. The model’s efficacy was evaluated using classification algorithms on the Nanfang and General Hospital MRI datasets (Kaggle). Among the algorithms tested, Random Forest demonstrated superior performance, achieving the highest accuracy and clinical utility (98.53% accuracy, 98.53% sensitivity, 99.51% specificity) compared to other machine learning algorithms, including SVM (97.22% accuracy), KNN (97.50% accuracy), and Naive Bayes (96.67% accuracy). This research underscores that Random Forest provides the most effective and clinically pertinent approach for precise brain tumor classification.

The study^32^ introduces a deep learning-based approach for predicting five critical genetic markers associated with glioma prognosis (IDH, 1p/19q codeletion, ATRX, MGMT, and TERT) from Whole Slide Images. The method derives its efficacy from a novel composite loss function, which integrates multi-label weighted cross-entropy loss for individual marker prediction, conditional probability loss for pairwise combinations, and spectral graph loss to account for collective group behaviors. The experimental results, corroborated by an ablation study, indicate that our approach achieves state-of-the-art predictive capability, markedly enhancing the accuracy of glioma biomarker prediction for prognostic and treatment planning purposes.

The research^33^ introduces DenseNet169-LIME-Tumor Net, a model that integrates DenseNet169 with LIME to evaluate the accuracy and interpretability of brain tumor classification. Trained on the Brain Tumor MRI Dataset comprising 2870 images, the model achieved an accuracy of 98.78%, outperforming Inception V3, ResNet50, MobileNet V2, and EfficientNet. LIME offers transparent visual explanations of predictions, facilitating clinical decision-making. The model’s low computational demands render it suitable for resource-constrained environments. Future research will expand the model to incorporate multi-modal learning and hybrid deep learning to enhance generalization in clinical settings.

A Dynamic Language Fusion (DLF) framework^28^ integrating ResNet18, LSTM for tumor evolution, BioGPT and BERT for clinical text, and cross-modal attention for feature fusion was validated on 10,287 MRI images from four datasets, achieving an accuracy of 98.96%, precision of 99.00%, and AUC above 0.998, outperforming previous models. The DLF framework address boundary ambiguity of neoplasms and feature overlap, while showing how temporal modeling and semantic understanding improve diagnostic performance. This study^34^ contributes a significant framework utilizing MRI image analysis for automated brain tumor diagnosis. This framework comprises the undecimated wavelet transform for frequency and temporal features extraction, EfficientNet-B0 for feature representation, principal component analysis (PCA) for feature selection, and a multi-support vector machine (multi-SVM) for classification. Classification is a 4-way problem distinguishing between meningioma, glioma, pituitary tumors, and non-tumor classes, ultimately achieving an overall accuracy of 97.5%. Development of this framework is an important step forward in advancing MRI-based brain tumor classification systems.

The study^45^ presented a new architecture with a Swin Transformer V2 based architecture for the automated classification of brain tumors from MRI scans. The main contributions include a Dual-Branch Down-sampling module and a Modified Attention Mechanism to improve multi-scale feature representation and computation performance. The model was trained on a dataset of 7023 grayscale MRI images and we achieved a state-of-the-art accuracy of 98.97%, which outperformed ResNet50 (90.39%) and DenseNet121 (93.20%). The model also achieved precision, recall, and F1-scores above 98% for each tumor class giving validation of robust and efficient abilities for brain tumor classification.

**Table 1:** Summary of brain tumor classification studies.

Ref.	Datasets used	Methodology utilized	Performance metrics	Research gaps
^35^	MRI scans of brain tumors	EfficientNetB7 withTL, channel attention mechanism	Superior performance in multiclass (accuracy) and binary classification (accuracy = 98%)	Need for implementation in clinical settings and refinement of validation methods.

^36^	Kaggle MRI dataset	SSD-based model with MobileNetV2 for tumor detection in low-resolution MRI scans	Accuracy = 98%, sensitivity and specificity not specified	Limited dataset size, lack of extensive clinical validation.

^37^	BraTS 2021, Br35H, BraTS 2023 datasets	Multi-grade hierarchical classification using Adaptive Hierarchical Optimized Horse Herd BiLSTM Fusion Network (AHOHH-BiLSTM)	Precision, recall, F1-score, and performance on BraTS datasets	Limited focus on non-glioma tumor classification, need for more diverse datasets for broader applicability.

^38^	MRI scans of brain tumors	ResNet101 with channel-wise attention mechanism (CWAM)	Accuracy = 99.83%, Recall = 99.21%, Precision = 99.01%, F1-score = 99.27%, AUC = 99.16%	Computational complexity due to CWAM, needs further model optimization for real-world deployment.

^39^	Kaggle datasets	VGG19 and ResNet50	Accuracy = 94% via VGG19, Accuracy = 93.5% via ResNet50	Need for implementation in clinical settings.

^40^	Two different datasets from Kaggle	End to end learning CNN	Accuracy = 96.86% for multi-class classification	Lacks in detailed refinement to classify other types of tumors.

^41^	Kaggle dataset + BraTS 2021 datasets of 8232 images	YOLOv7 +TL	Accuracy = 99% for diagnosis viaTL	Lacks in validation methods for extensive survey.

^42^	Kaggle dataset of 3000 images	Fine tuning of InceptionV3, VGG-19 and VGG-16	Accuracy = 89% for InceptionV3, Accuracy = 96% for VGG16, and Accuracy = 98% for VGG19	Lacks in segmentation which helps in tumor detection.

^35^	MR scans of brain tumors	TL-based optimized ResNet152 model	Accuracy = 98.53%, F1 score = 97.4%, Sensitivity = 96.52%	Inconsistent image sizes, overfitting in ML models, lack of comprehensive classifier comparison, and insufficient comparison between deep learning and traditional methods.

^43^	MBTD, BraTS 2020 dataset	ARM-Net: Attention-guided residual multiscale CNN	Accuracy = 96.64% (MBTD), 97.11% (BraTS 2020)	Difficulty in capturing subtle lesion size/shape variations, and CNN models’ inefficiency in capturing multiscale features.

^44^	Kaggle dataset + additional datasets	Multi-path CNN with SVM classifier	Accuracy = 98.3% (First dataset), 98.2% (Second dataset), 99.1% (combined)	Need for better classifier alternatives, and issues with CNN-SVM performance in multi-class classification.

^45^	CE-MRI, TT-MRI (Brain tumor MRI datasets)	TL with Swin Transformer model (SwinBTC), data augmentation	Accuracy = 98.53%, F1 score = 97.4%, Sensitivity = 96.52%	Inconsistent image sizes, overfitting, and limited comparison of deep learning vs traditional methods.

^46^	Kaggle dataset of 7023 grayscale MRI images	Improved Swin Transformer V2 with Dual-Branch Down-sampling module and Enhanced Attention Mechanism	Accuracy = 98.97%, Precision = 98.75%, Recall = 98.51%, F1 score = 98.63%	Difficulty in handling small tumors, misclassification of pituitary tumors, and the need for multi-modal input enhancement.

The study^47^ introduces the FHAT_EfficientNet model for multi-grade brain tumor classification utilizing federated learning. The FHAT_EfficientNet model incorporates fractional calculus (FC) and the harmony search-based feedback artificial tree (HSFAT) algorithm, which integrates harmony search with feedback artificial tree. The methodology involved pre-processing MRI images, performing tumor segmentations using fuzzy local information c-means (FLICM), and applying image augmentations and feature extraction. The model, fine-tuned with EfficientNet, demonstrated outstanding performance, achieving 91.7% accuracy, 93.6% specificity, 96.6% sensitivity, MSE (0.058), RMSE (0.241), and loss (0.083).

The integration of SwinBTC,^48^ a brain tumor classification model employing a pretrained Swin Transformer within aTL framework to enhance brain tumor detection from MRI scans. The model utilizes clinical MRI data from the CE-MRI and TT-MRI datasets, incorporating both online and offline augmentations to enhance generalization and mitigate overfitting. The Swin Transformer’s gradient-based hierarchical self-attention mechanism effectively captures both global and local contexts, thereby improving training convergence and reducing overfitting. Experimental results indicate that Swin BTC surpasses previous baseline models, offering more precise tumor classification, which is crucial for accurate treatment planning.

Research on brain tumor detection has identified several limitations in existing methodologies, including inconsistent input image sizes and a limited combination of dataset diversity and data range variations, often leading to inaccurate tumor detection.^49^ Many existing models tend to overfit relatively small, over-specialized datasets, thereby limiting their ability to generalize to new and unseen data.^50^ Furthermore, traditional computerized methods that rely on handcrafted features are inherently incapable of effectively modeling the complex, high-dimensional patterns present in MRI images.^51^ Although deep learning is often regarded as the pinnacle of model performance, contemporary models have not been sufficiently contrasted with non-trained data as conventional models have been. Without comprehensive practical application, it remains challenging to identify the optimal model for brain tumor classification. Indeed, performance metrics reported in earlier studies are frequently misrepresented, leading to inaccurate performance evaluations. A more effective model, which integrates advanced pre-processing, dimensionality reduction, data augmentation, and feature extraction methods, along with classifier comparisons, is likely to yield a more reliable and accurate solution for clinical brain tumor detection. The Table 1 illustrates the overview of existing studies that contain limitations and gaps in brain tumor prediction.

**Fig. 1:**
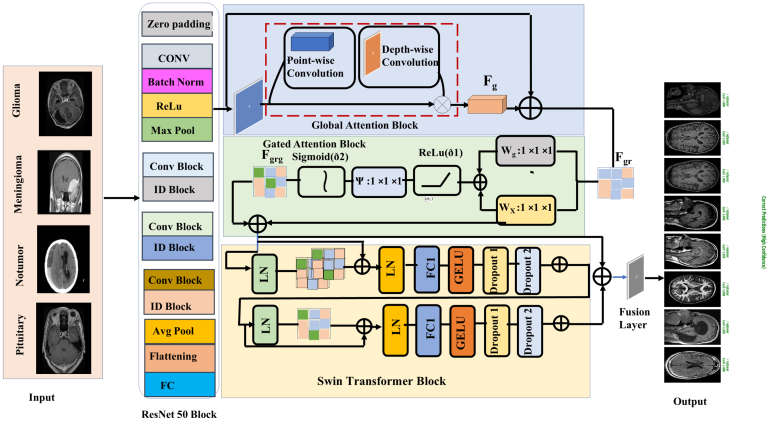
Schematic representation of the ResSGA-Net framework, integrating ResNet50, dual attention mechanisms, and Swin Transformer with Fusion Block.

## 3 Methodology

The **ResSGA-Net** architecture integrates a **ResNet50 backbone**, dual-attention mechanisms, a **Swin Transformer**, and a **Fusion Block** for brain tumor classification from MRI images. The model extracts multi-scale features, refines them using attention mechanisms, and captures both local and global contexts through the Swin Transformer. A schematic of the architecture is shown in Fig. 1.

The ResNet50 backbone is used for feature extraction, capturing hierarchical image features through its deep architecture. The input MRI image $I\in R^{H\times W\times C}$, where H and W are the image dimensions and C is the number of channels (typically 1 for grayscale MRI), is first processed through an initial convolutional layer followed by batch normalization (BN) and ReLU activation. These operations extract low-level features, such as edges and textures, which are essential for distinguishing tumor regions.

The core of **ResNet50** consists of **residual connections**, which allow the network to learn deep representations without the vanishing gradient problem. These connections enable the network to preserve essential spatial hierarchies, which are critical for tumor detection. The residual learning can be expressed as:

$$X_{i}=F\left(X_{i-1}\right)+X_{i-1}$$

where F(⋅) represents the residual function (convolution, batch normalization, and ReLU), and $X_{i-1}$ is the feature map input to the residual block.

After the residual blocks, **Global Average Pooling (GAP)** aggregates the features into a compact representation:

$$X_{\text{GAP}}=\frac{1}{H\times W}\overset{H}{\underset{i=1}{\sum }}\overset{W}{\underset{j=1}{\sum }}X_{i}$$

where H and W are the height and width of the feature map, and $X_{i}$ is the feature map value. GAP provides a global summary of the spatial features extracted from the image.

### 3.1 Attention mechanisms

To refine the feature maps from **ResNet50**, the model incorporates a **Global Attention Mechanism (GAM)** and a **Gated Attention Mechanism (GAM)**. The **Global Attention Mechanism** captures long-range dependencies across the feature map, allowing the model to focus on critical tumor regions while suppressing irrelevant background. This is expressed as:

$$A=\text{Softmax}\left(QK^{T}\right)$$

where Q and K represent the query and key matrices, and A is the attention map that highlights important regions such as the tumor area.

The **Gated Attention Mechanism** refines the feature maps further by applying a gating function that selectively emphasizes the most relevant features, allowing the model to prioritize tumor-related areas while suppressing background information. The gated feature map is computed as:

$$F_{\text{gated}}=\overset{ˆ}{g}⊙F_{\text{gr}}$$

where $\overset{ˆ}{g}$ is the gating mask, and ⊙ represents element-wise multiplication.

### 3.2 Swin transformer

**Fig. 2:**
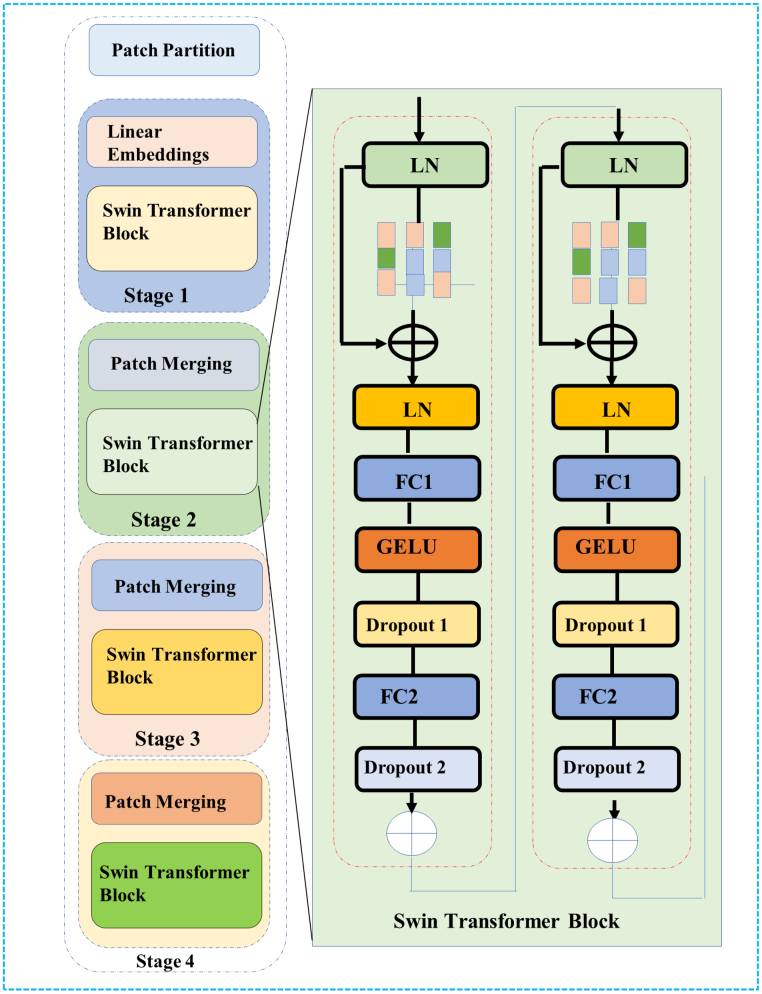
Swin Transformer framework showing patch partitioning, merging, and block composition.

The **Swin Transformer** is integrated to capture both **local** and **global contextual relationships**. The transformer divides the image into non-overlapping patches and processes them in a hierarchical manner, capturing fine-grained local features and larger, contextual patterns. The output feature map $F_{\text{Swin}}\in R^{C_{\text{out}}\times H\times W}$ is computed as:

$$F_{\text{Swin}}=\text{SwinTransformer}\left(F_{\text{grg}}\right)$$

where $F_{\text{grg}}$ is the concatenated feature map from the attention mechanisms and ResNet50. This transformer enhances the model’s ability to capture complex tumor structures and improves the network’s generalization ability across different tumor types (see Fig. 2).

### 3.3 Fusion block

The **Fusion Block** integrates the outputs from **ResNet50**, **attention mechanisms**, and the **Swin Transformer**. This block combines multi-scale convolutional features with attention-refined and transformer-enhanced representations to create a unified feature map, which is then used for classification. The fusion operation is mathematically expressed as:

$$F_{\text{fusion}}=F_{\text{drop1}}+F_{\text{drop2}}$$

where $F_{\text{drop1}}$ and $F_{\text{drop2}}$ represent the outputs from the two parallel processing paths. The Fusion Block ensures that the model benefits from the strengths of each component for accurate classification.

### 3.4 Design rationale and architectural justification

Although ResSGA-Net integrates multiple well-established components, its contribution lies in the principled orchestration and interaction of these modules rather than their standalone use. Each architectural component addresses a distinct and complementary limitation observed in brain tumor MRI classification, and their integration follows a deliberate design logic rather than architectural aggregation.

The ResNet50 backbone is employed as a stable hierarchical feature extractor, leveraging residual learning to preserve low- and mid-level anatomical features that are critical for tumor localization. However, convolutional backbones alone are limited in modeling long-range spatial dependencies and often struggle with heterogeneous tumor boundaries.

To address this limitation, global attention is introduced immediately after convolutional feature extraction to enhance inter-regional dependency modeling across the entire feature map. This enables the network to capture contextual relationships between spatially distant tumor regions that cannot be reliably modeled by convolution alone. Subsequently, gated attention is applied to suppress irrelevant activations and selectively amplify tumor-relevant responses, thereby improving feature discriminability under class overlap conditions.

The Swin Transformer is intentionally placed after attention refinement rather than directly on raw feature maps. This design choice ensures that the transformer operates on semantically enriched and noise-suppressed representations, allowing it to model hierarchical contextual relationships without being overwhelmed by irrelevant low-level variations. This sequential refinement contrasts with parallel or early-fusion transformer designs commonly adopted in existing hybrid architectures.

Finally, the fusion mechanism consolidates convolutional locality, attention-enhanced saliency, and transformer-based global context into a unified representation. This structured interaction enables ResSGA-Net to balance local detail preservation with global contextual awareness, which is essential for robust multi-class tumor discrimination.

## 4 Results and experiment

All experiments, including the main evaluation and ablation studies, were conducted on benchmark brain tumor MRI datasets. The proposed attention-enhanced framework consistently outperformed the baseline ResNet50 model across all evaluation metrics, demonstrating its superior classification capability. The ablation results further verified the individual and collective contributions of the Gated Attention, Global Attention, and Swin Transformer modules, each of which enhanced model performance when integrated into the architecture. These findings confirm that the proposed framework is both robust and effective, offering reliable improvements in brain tumor classification.

### 4.1 Dataset description

**Dataset I:** The dataset employed in this study is publicly available on Kaggle.^f^ It contains brain MRI scans categorized into four classes: glioma tumor, meningioma tumor, pituitary tumor, and normal. The dataset provides a balanced collection of images with sufficient diversity in tumor types, sizes, and anatomical locations, enabling reliable training and evaluation of classification models. Its open accessibility also ensures reproducibility and facilitates comparative analysis with other state-of-the-art approaches.

**Dataset II:** The second dataset used in this study is publicly available on Kaggle^g^ and is commonly referred to as the Figshare Brain Tumor Classification dataset. It consists of T1-weighted contrast-enhanced brain MRI scans categorized into three tumor classes: glioma, meningioma, and pituitary tumor. The dataset contains a diverse set of images acquired from different patients, exhibiting variations in tumor morphology, size, location, and imaging conditions. This diversity makes the dataset well suited for evaluating the robustness and generalization capability of multi-class brain tumor classification models. Its public availability and widespread use in the literature further support reproducibility and enable fair comparison with existing state-of-the-art methods.

### 4.2 Implementation details

The proposed network was implemented in PyTorch and trained in an end-to-end fashion on the brain tumor MRI datasets. For evaluation we adopted a stratified 3-fold cross-validation (CV) method to ensure that all classes were properly represented across folds. A Weighted Random Sampler was utilized during training to accounts for class imbalance. An input MRI scan was resized to 224 × 224 pixels and normalized according to ImageNet statistics. Many augmentation techniques, including random horizontal (p = 0.5), vertical (p = 0.3), random rotation (±30°), random cropping, color jitter (brightness/contrast/saturation/hue), grayscale, posterization, solarization, random erasing, and so on, were also used to minimize overfitting and improve generalization. Performing augmentation techniques abstracts the data and enhances robustness with respect to variations in imaging.

The networks were trained for 50 epochs (batch size 16) including the proposed attention-enhanced ResNet50 backbone with local, gated, global, and Swin Transformer modules. The AdamW optimizer (α=0.9, β=0.999, weight decay = 0.005, $\epsilon =1\times 10^{-7}$) was used with an initial learning rate of $1\times 10^{-4}$. The learning rate followed a warm-up cosine decay schedule, and training utilized mixed-precision (AMP) and gradient clipping (max norm = 0.5) for stability and efficiency. The cross-entropy loss function was used for optimization. All experimental results were generated on NVIDIA GPUs with CUDA.

### 4.3 Performance analysis using dataset I

The collective classification report of the three folds clearly indicates that the proposed ResSGA-Net model performed excellently in all classifications of brain tumor patients. The “No Tumor” class had the most overall performance as illustrated in Table 2 with nearly perfect scores (Precision = 0.99, Recall = 0.99, F1 = 0.99); the high scores suggest the model’s ability to differentiate healthy cases where getting false positives is very low. The Glioma and Meningioma classes also demonstrated reliable performances with F1-scores of 0.98 and 0.98, respectively, indicating that there were similar scores in terms of precision and recall across the two tumor types. The Pituitary class (Precision = 1.00) had perfect precision where the model appropriately assigned positive cases and did not misclassify other tumor classes as Pituitary however the recall (0.95) was less than perfect suggesting that there was slight misclassification in the number of pituitary tumors that were incorrectly categorized. Based on the overall results it is confirmed that the model has consistent performances that are suitable across all tumor categories and confirms the diagnostic capability and efficacy to apply clinically.

The fold-wise outcomes of ResSGA-Net confirm that the results are stable and consistent over the tumor class outcomes as shown in Table 3. “No Tumor” had the most stable results (F1-scores below 0.9993) in tracking responses for patients with and without the glioma, indicating clear distinctions between the glioma characterization (F1 > 0.98 over folds), while meningoma consistently saw very high recall approaching 1.0000. Pituitary class had consistently strong precision (> 0.99) but variable recalls through different folds. Overall the results serve as a validation study for proposed architecture to result outcomes that are stable over multiple cross-validations.

**Table 2:** Combined classification report.

Class	Precision	Recall	F1-score
Glioma	0.97	0.99	0.98
Meningioma	0.96	0.99	0.97
No Tumor	0.99	0.99	0.99
Pituitary	1.00	0.95	0.97

**Fig. 3:**
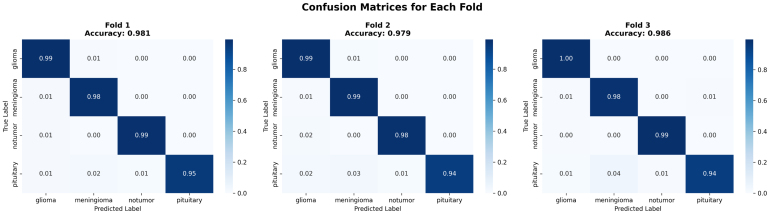
Confusion matrices for each fold of the model validation .

Across the three folds, the confusion matrices in Fig. 3 present the performance and stability of the proposed ResSGA-Net as sufficient and consistent. For example, in Fold 1 (accuracy: 0.981), the majority of classes classified almost perfectly with only quite minor misclassifications noted only in confusions between pituitary and no tumor. Similar performance was observed in fold 2 (accuracy: 0.979) but pituitary, again, showed decreased performance with some overlap with the no tumor predictions preventing the models achieving the higher values. In Fold 3 (accuracy: 0.986), we achieved the highest overall accuracy where glioma and no tumor were classified almost perfectly and only three notable errors in pituitary predictions. Overall, glioma and meningioma had strong recognition rates in every fold, predominantly above 0.98, while the no tumor class has a normalized data reach above 0.99. The pituitary class achieved particularly high precision rates but achieved a lower recall rate due to slightly confusing classifications in a small number of instances. Such results confirm that the framework generalizes well across folds, with stable classification performance but also provide an opportunity to improve pituitary tumor recognition.

**Table 3:** Fold-wise classification report of proposed model (ResSGA-Net).

Tumor class	Fold	Precision	Recall	F1-score
Meningioma	Fold-1	0.9437	0.9991	0.9718
	Fold-2	0.9478	0.9892	0.9663
	Fold-3	0.9721	0.9814	0.9767

Glioma	Fold-1	0.9812	0.9819	0.9815
	Fold-2	0.9745	0.9923	0.9833
	Fold-3	0.9767	0.9991	0.9878

Pituitary	Fold-1	1.0000	0.9524	0.9756
	Fold-2	0.9988	0.9321	0.9642
	Fold-3	0.9913	0.9498	0.9701

No Tumor	Fold-1	0.9917	0.9912	0.9914
	Fold-2	0.9945	0.9991	0.9968
	Fold-3	0.9992	0.9994	0.9993

**Fig. 4:**
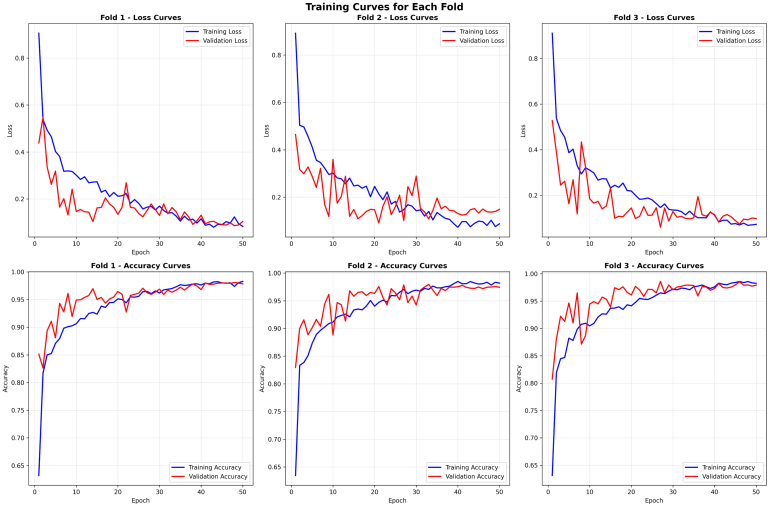
Training and validation loss and accuracy curves for each fold during model training .

The training and validation curves across all three folds demonstrated in Fig. 4 stable convergence for the proposed classifier, which confirms both effective learning and strong generalization. In terms of loss curves, training loss consistently decreased with epochs, while validation loss exhibited similar behavior, indicating no major overfitting issues. These curves experienced some unpredictable behavior, but the overall outcome is more stable by around the 40th epoch. With regards to accuracy curves, both training and validation accuracy made rapid increases in the first 20 epochs before gradually reaching a plateau of above 0.97. Validation accuracy steadily converged with training accuracy, demonstrating that the model generalizes well to new, unseen data - fold-wise - across the learning phase. The consistent behavior is evidence that the optimization process worked effectively, and the utilization of managed augmentation, sampling strategies, and attention modules influenced consistent performance across folds.

**Fig. 5:**
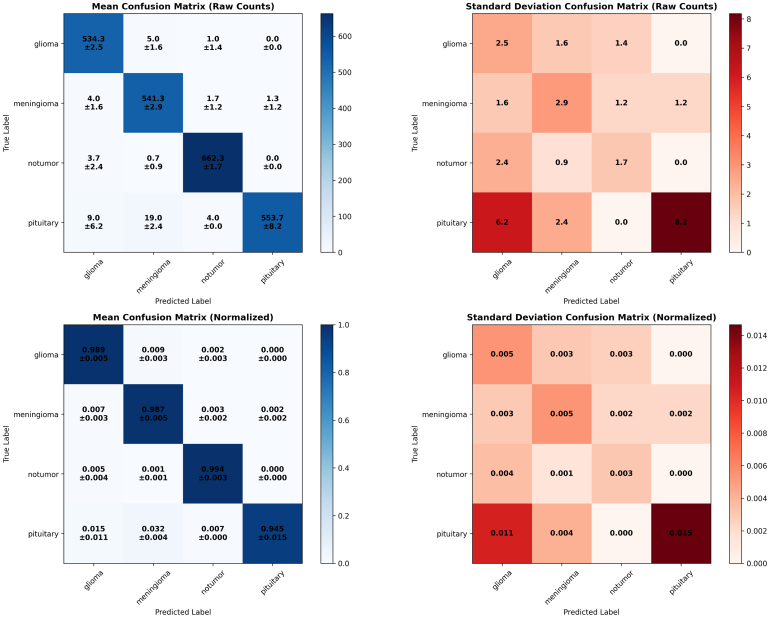
Confusion matrices (raw counts and normalized) with standard deviations for glioma, meningioma, notumor, and pituitary classifications .

The overall confusion matrices give further insight into the consistency and fidelity of the ResSGA-Net model across folds as well illustrated in Fig. 5. The mean confusion matrix (raw counts and normalized) indicates that the ResSGA-Net correctly classifies the vast majority of samples in all classes with normalized accuracies greater than .98 for glioma, meningioma, and no tumor and .945 for pituitary. Misclassification is low, and only really occurs between pituitary and meningioma or pituitary and glioma, which is expected considering the structure of certain MRI slices. The standard deviation matrices also show this in terms of prediction variability; the variability across folds was quite low (less than .005-.015 in terms of normalized values). The main difference is the class for pituitary, which had slightly higher variances across folds when compared to the other classes indicating the model occasionally confuses pituitary with the other adjacent class categories. Overall, these results show the ResSGA-Net model is accurately and consistently predicting regional image class samples across each split of the cross-validation, with variation for pituitary values only.

**Fig. 6:**
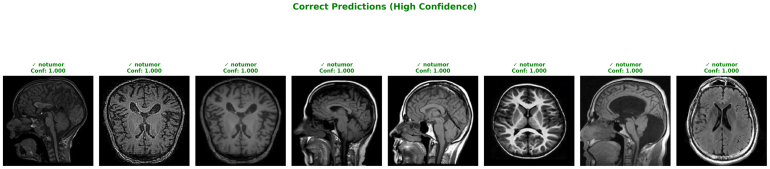
High confidence correct predictions for ‘No Tumor’ classifications .

The first visualization highlights examples of correct predictions made by the proposed model with extremely high confidence (confidence score = 1.000). All of the selected cases shown in Fig. 6 belong to the “No Tumor” class, where the model demonstrates complete certainty in classification. This strong performance emphasizes the model’s ability to accurately distinguish healthy brain MRI scans from abnormal cases, a crucial factor in clinical screening applications. The consistency in correct, high-confidence predictions across multiple patients validates the robustness of the feature extraction and attention modules, particularly in detecting the absence of pathology where false positives must be minimized.

**Fig. 7:**
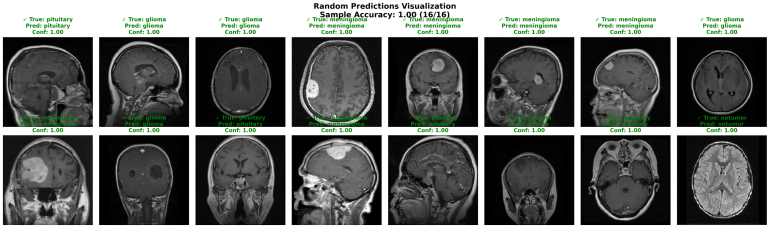
Visualization of random predictions for brain tumor classification with 100% accuracy across various tumor types (glioma, meningioma, pituitary, and non-tumor).

The visualization illustrates in Fig. 7 random predictions across different tumor classes (glioma, meningioma, pituitary) and no tumor cases, where the model again achieved 100% sample accuracy (16/16) with confidence scores of 1.000. The Figure shows that the network correctly identifies tumors of varying shapes, sizes, and locations across patients, highlighting its adaptability to inter-patient variability. Importantly, predictions remain reliable even in challenging cases such as large pituitary masses or subtle glioma regions. This demonstrates the model’s capability not only in general classification but also in handling diverse tumor presentations. Together, these results confirm that the proposed framework provides both accuracy and interpretability, which are critical for deployment in real-world clinical decision support systems

**Fig. 8:**
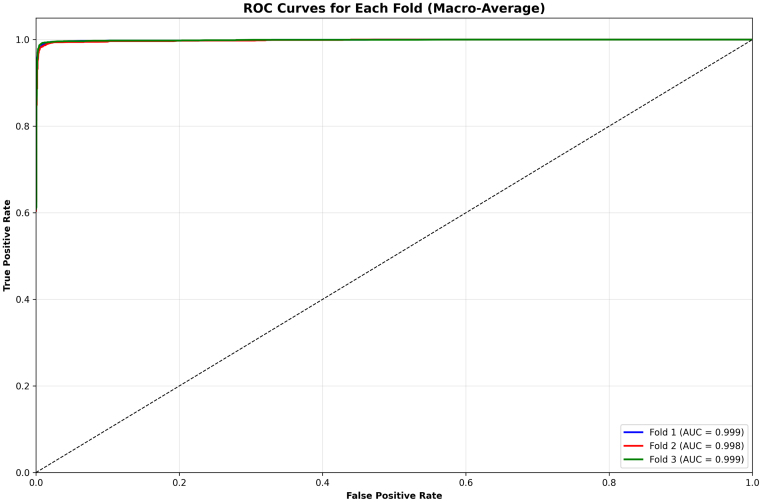
ROC curves for each fold with macro-average AUC scores .

The macro-averaged ROC curves across the three folds demonstrates in Fig. 8 the remarkable discriminative power of the proposed ResSGA-Net. All folds achieve nearly perfect outcomes with AUC values of 0.999, 0.998 and 0.999 for Fold 1, 2 and 3 respectively. The curves trail closely along the top-left of the plot, indicative of an outstanding trade-off between sensitivity and specificity. The reliable consistency across the three folds in the AUC values indicates that the model generalizes well and consistently performs well ignoring the specific data splits. These results indicate that the framework is robust in discriminating cases of tumors from none and at the same time reduced the incidence of false positives and false negatives.

**Fig. 9:**
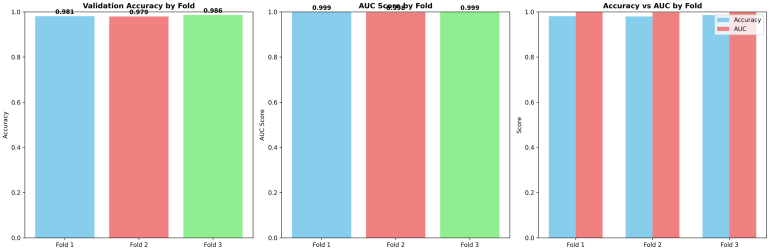
Comparison of validation accuracy, AUC scores, and accuracy vs AUC by fold in 5-fold cross-validation.

The fold-wise summaries of validation accuracy and AUC in Fig. 9 provide added validation of the stability and reliability of the proposed ResSGA-Net. The validation accuracy was consistently high for all folds and ranged between 0.979 and 0.986, while the AUC values were nearly perfect at 0.999 in every instance of a fold. The comparative visualization of accuracy and AUC in Fig. 3D showed a strong relationship between the two measures which further reinforces that the model achieved high classification accuracy and effectively discriminated between positive and negative cases across all tumor classes. Overall, these results provide compelling evidence for the excellent generalization of the framework and its use-case for diagnostic applications in reality.

## 5 Performance analysis using dataset II

This section presents a detailed evaluation of the proposed model on **Dataset II**, covering overall classification performance, class-wise behavior, robustness under cross-validation, fold-wise consistency, and statistical significance. Dataset II contains three tumor categories: glioma, meningioma, and pituitary tumor.

### 5.1 Overall classification performance

Table 4 summarizes the classification performance obtained on Dataset II. The proposed model achieves an overall accuracy of **92%**, indicating strong discriminative capability across all tumor classes. Both macro-averaged and weighted-averaged F1-scores reach **0.92**, confirming balanced performance despite class imbalance.

Glioma cases exhibit high precision (0.97), indicating reliable predictions, while recall (0.87) is comparatively lower, reflecting the heterogeneous and infiltrative nature of glioma tumors. Meningioma achieves the most balanced performance, with precision and recall values of 0.92 and 0.97, respectively, resulting in the highest F1-score (0.94). Pituitary tumors demonstrate high recall (0.97), indicating strong sensitivity, although precision is slightly reduced (0.87), likely due to visual similarity with adjacent anatomical structures.

**Table 4:** Classification performance on Dataset II.

Class	Precision	Recall	F1-score	Support
Glioma	0.97	0.87	0.92	475
Meningioma	0.92	0.97	0.94	236
Pituitary tumor	0.87	0.97	0.92	310

Accuracy	0.92 (1021 samples)			
Macro Avg	0.92	0.94	0.92	1021
Weighted Avg	0.93	0.92	0.92	1021

### 5.2 Cross-validation performance and stability

To evaluate robustness and generalization capability, a three-fold cross-validation strategy was employed. Table 5 reports the mean and standard deviation across folds. The model achieves a mean accuracy of 0.9318±0.0080 and an AUC score of 0.9893±0.0020, demonstrating excellent class separability.

The low standard deviation across all metrics indicates stable learning behavior and minimal sensitivity to data partitioning. The macro-averaged F1-score of 0.9260±0.0046 further confirms balanced multi-class performance.

**Table 5:** Cross-validation performance on Dataset II (Mean ± STD).

Metric	Mean ± STD
Accuracy	0.9318±0.0080
AUC Score	0.9893±0.0020
Loss	0.2613±0.0229
Macro Precision	0.9216±0.0038
Macro Recall	0.9329±0.0059
Macro F1-score	0.9260±0.0046
Weighted Precision	0.9268±0.0037
Weighted Recall	0.9243±0.0046
Weighted F1-score	0.9242±0.0045

### 5.3 Per-class cross-validation analysis

Table 6 presents per-class performance averaged across folds. Meningioma consistently achieves the highest F1-score, indicating strong separability. Glioma and pituitary tumor classes maintain comparable F1-scores, reflecting balanced sensitivity and precision.

The small standard deviations observed for all classes demonstrate consistent behavior across folds, confirming that class-wise performance is not dependent on a specific data split.

**Table 6:** Per-class performance on Dataset II (Mean ± STD).

Class	Precision	Recall	F1-score	Support
Glioma	0.9530±0.0118	0.8899±0.0147	0.9202±0.0034	475.3
Meningioma	0.9206±0.0170	0.9562±0.0269	0.9376±0.0044	236.0
Pituitary tumor	0.8912±0.0309	0.9527±0.0191	0.9202±0.0072	310.0

**Fig. 10:**
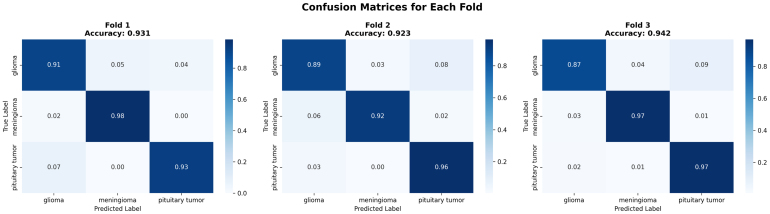
Fold-wise confusion matrices for Dataset II. Each matrix is row-normalized, where rows correspond to true labels and columns represent predicted labels. Diagonal entries indicate correct classifications, while off-diagonal values represent misclassification rates.

### 5.4 Fold-wise performance analysis

Table 7 reports individual fold results. Fold 1 and Fold 3 achieve higher accuracy and AUC scores, while Fold 2 exhibits slightly reduced performance. However, performance variation across folds remains limited, indicating robust generalization.

Fold 3 achieves the highest accuracy (0.9422) and AUC (0.9912), while Fold 2 represents the most challenging split. Importantly, macro-averaged metrics remain consistently high across all folds, confirming stability under varying training-validation distributions.

The fold-wise confusion matrices shown in Fig. 10 provide a detailed class-level interpretation of the quantitative results reported earlier in this section, including the classification metrics (Table 4), cross-validation summary (Table 5), and fold-wise performance analysis (Table 7).

**Table 7:** Fold-wise performance on Dataset II.

Fold	Accuracy	AUC	Loss	Macro precision	Macro recall	Macro F1
Fold 1	0.9305	0.9901	0.2291	0.9269	0.9388	0.9322
Fold 2	0.9226	0.9866	0.2741	0.9197	0.9248	0.9211
Fold 3	0.9422	0.9912	0.2807	0.9183	0.9351	0.9247

Consistent with the high overall accuracy and macro-averaged recall reported previously, all three confusion matrices exhibit strong diagonal dominance, indicating that the majority of samples are correctly classified across all folds. This observation directly supports the reported macro-average recall of 0.94 and the stable macro F1-score of 0.9260±0.0046, confirming balanced sensitivity across tumor classes.

In Fold 1, the model achieves correct classification rates of 91% for glioma, 98% for meningioma, and 93% for pituitary tumors. These results align with the fold-wise accuracy of 0.9305 and the strong per-class precision reported earlier. Minor confusion between glioma and pituitary tumors explains the slightly lower recall observed for glioma in the classification report, while the near-perfect meningioma recognition is consistent with its highest F1-score among all classes.

Fold 2 represents the most challenging validation split, as reflected by its comparatively lower accuracy (0.9226) and AUC score (0.9866). The confusion matrix reveals increased misclassification of glioma samples toward pituitary tumors, which explains the reduction in glioma recall and the slight increase in validation loss reported for this fold. Despite this, meningioma and pituitary tumor classes maintain high recognition rates (92% and 96%, respectively), supporting the robustness indicated by the weighted-average performance metrics.

**Fig. 11:**
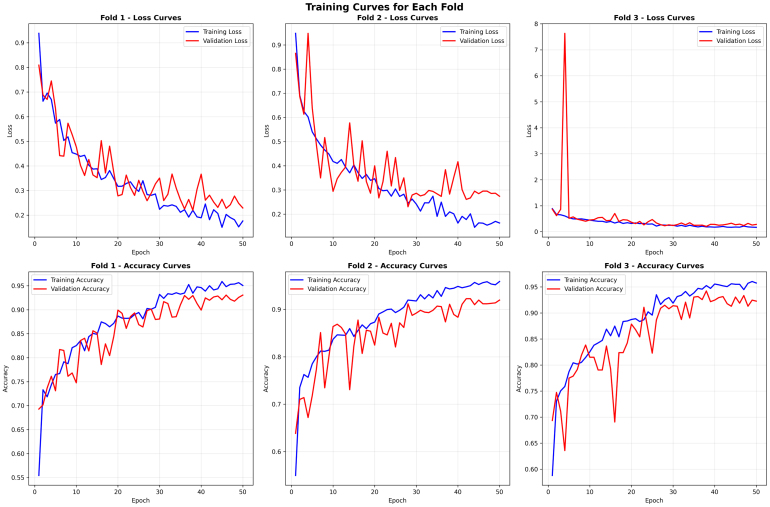
Training and validation loss and accuracy curves for each cross-validation fold on Dataset II. The curves illustrate optimization stability, convergence behavior, and generalization performance across folds.

**Fig. 12:**
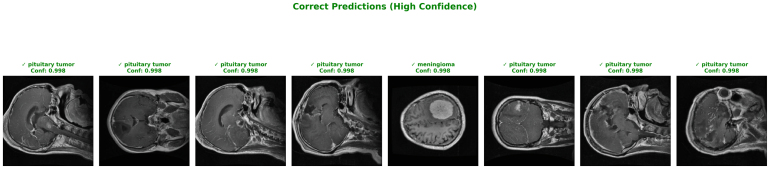
Representative examples of correctly classified MRI slices from Dataset II with high prediction confidence. The model assigns confidence scores close to 1.0 for these samples, illustrating strong class separability and reliable decision-making across tumor categories.

Fold 3 achieves the highest accuracy (0.9422) and demonstrates the most balanced confusion matrix. Meningioma and pituitary tumors both achieve 97% correct classification, while glioma maintains stable performance with limited misclassification. This behavior is consistent with the superior fold-wise accuracy and AUC score observed in Table 7, as well as the narrow confidence intervals reported in the statistical analysis.

Overall, the confusion matrices provide qualitative confirmation of the quantitative findings presented earlier. The limited off-diagonal entries correspond directly to the small standard deviations observed in cross-validation metrics, while the consistent class-wise behavior across folds supports the statistical significance and large effect sizes reported. Together, these results demonstrate that the proposed model achieves stable, reliable, and clinically meaningful multi-class brain tumor classification performance on Dataset II.

Fig. 11 illustrates the training and validation loss and accuracy curves for each fold during three-fold cross-validation on Dataset II. These curves provide insight into the optimization dynamics and explain the fold-wise performance trends reported earlier.

In Fold 1, both training and validation losses decrease steadily, while accuracy curves exhibit consistent improvement with a minimal generalization gap. This stable convergence behavior explains the strong fold-wise accuracy and the clean confusion matrix observed for this fold, particularly the near-perfect recognition of meningioma cases.

Fold 2 exhibits larger fluctuations in validation loss and accuracy, especially during early training stages, indicating higher data variability and class overlap. This behavior aligns with the slightly reduced accuracy and increased glioma–pituitary misclassification observed in the corresponding confusion matrix. Nevertheless, the convergence of training and validation curves toward later epochs confirms that the model remains stable and avoids overfitting.

Fold 3 demonstrates the most stable and well-aligned convergence behavior, with rapid loss reduction and smooth accuracy improvement. The close alignment between training and validation curves indicates strong generalization, directly supporting the highest fold-wise accuracy and balanced confusion matrix reported earlier.

Overall, the convergence patterns across all folds confirm stable optimization and robust generalization. The consistency between training dynamics, confusion matrices, and cross-validation metrics reinforces the reliability and statistical significance of the proposed model for multi-class brain tumor classification.

Fig. 12 presents representative examples of correctly classified MRI slices from Dataset II with high prediction confidence. These qualitative results provide visual confirmation of the strong quantitative performance reported earlier.

The confidence scores approaching 1.0 are consistent with the high AUC value and the strong diagonal dominance observed in the confusion matrices across all folds. Most high-confidence predictions correspond to pituitary tumors, aligning with the consistently high recall values reported for this class in both the classification report and cross-validation analysis. Correctly classified meningioma samples with similarly high confidence further support the balanced class-wise performance observed in earlier results.

The presence of high-confidence predictions across diverse anatomical views and imaging conditions indicates that the model has learned robust and discriminative representations rather than relying on dataset-specific artifacts. This behavior is further supported by the stable training and validation convergence patterns, which showed no evidence of overfitting.

**Fig. 13:**
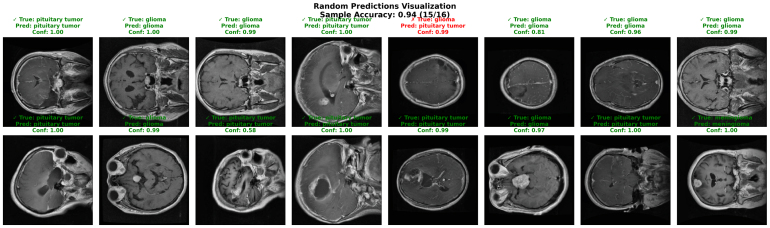
Random prediction visualization on Dataset II. Each sample shows the ground-truth label, predicted class, and associated confidence score. Correct predictions are marked in green, while misclassified samples are highlighted in red.

Taken together, these qualitative examples complement the quantitative metrics, confusion matrix analysis, and statistical significance testing, demonstrating that the proposed model achieves reliable, well-calibrated, and clinically meaningful predictions on Dataset II. Fig. 13 presents randomly selected test samples from Dataset II along with their predicted labels and confidence scores. Unlike curated examples, this visualization provides an unbiased qualitative assessment of model behavior in realistic inference scenarios.

The reported sample accuracy of 0.94 (15/16) closely aligns with the cross-validation accuracy and weighted F1-score reported earlier, confirming consistency between qualitative and quantitative evaluations. Most samples are correctly classified with high confidence, which is consistent with the high AUC score and strong diagonal dominance observed in the confusion matrices.

The visualization includes a single misclassified case, where a glioma sample is predicted as a pituitary tumor. This error pattern is consistent with the confusion matrix analysis, which identified glioma–pituitary tumor confusion as the primary source of misclassification. Such cases typically involve ambiguous tumor morphology and overlapping intensity characteristics.

Additionally, the presence of correct predictions with moderate confidence values indicates that the model does not produce uniformly overconfident outputs, suggesting reasonable confidence calibration. This behavior aligns with the stable convergence observed in the training and validation curves and supports the reliability of the statistical significance analysis.

**Fig. 14:**
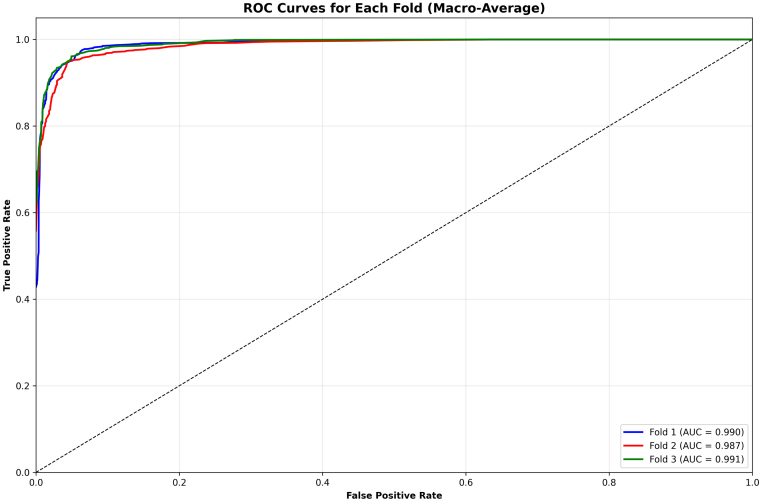
Macro-averaged ROC curves for each cross-validation fold on Dataset II. The curves demonstrate strong discriminative performance across tumor classes, with consistently high AUC values across folds.

Overall, this random prediction analysis confirms that the proposed model maintains robust, generalizable, and well-calibrated performance across diverse test samples, reinforcing its suitability for practical brain tumor classification tasks. Fig. 14 illustrates the macro-averaged ROC curves obtained across three cross-validation folds on Dataset II. All folds achieve near-perfect ROC characteristics, with AUC values exceeding 0.98, indicating excellent class separability.

Fold 1 achieves an AUC of 0.990, consistent with its strong fold-wise accuracy and minimal inter-class confusion observed in the corresponding confusion matrix. Fold 2 exhibits a slightly reduced AUC of 0.987, reflecting the increased glioma–pituitary tumor confusion and higher validation loss fluctuations reported earlier. Despite this, the ROC curve remains steep at low false positive rates, confirming robust discriminative capability.

Fold 3 achieves the highest AUC of 0.991, aligning with its superior fold-wise accuracy, stable training convergence, and highly balanced confusion matrix. The close overlap of ROC curves across folds indicates low variance and strong generalization, supporting the narrow confidence intervals and large effect sizes reported in the statistical significance analysis.

Overall, the macro-averaged ROC analysis complements the classification metrics, confusion matrix interpretation, training dynamics, and qualitative visualizations, confirming that the proposed model maintains stable and reliable discriminative performance across different data partitions.

**Fig. 15:**
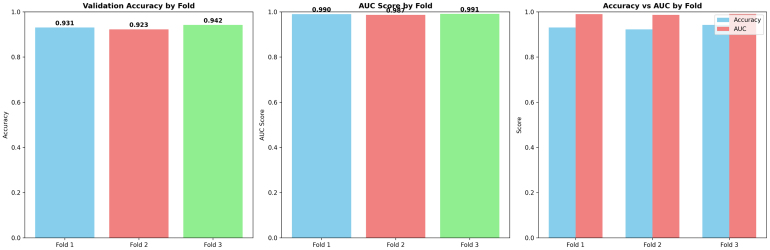
Fold-wise comparison of validation accuracy and AUC scores on Dataset II. The plots highlight consistent performance across folds and illustrate the relationship between point-estimate accuracy and threshold-independent discriminative ability.

Fig. 15 presents a fold-wise comparison of validation accuracy and AUC scores on Dataset II. Across all folds, both metrics remain consistently high, indicating stable generalization and strong discriminative capability.

Fold 1 achieves a validation accuracy of 0.931 and an AUC of 0.990, consistent with its stable training convergence, clean confusion matrix, and steep ROC curve. Fold 2 exhibits a slight reduction in both accuracy (0.923) and AUC (0.987), reflecting increased data complexity and higher glioma–pituitary tumor confusion observed earlier. Nevertheless, the AUC remains near-perfect, confirming that reduced accuracy is primarily due to threshold-specific effects rather than poor class separability.

Fold 3 attains the highest validation accuracy (0.942) and AUC (0.991), aligning with its superior training stability, balanced confusion matrix, and strongest ROC performance. The minimal variation across folds supports the low standard deviations and narrow confidence intervals reported in the cross-validation and statistical significance analyses.

Overall, this comparison reinforces that the proposed model achieves a favorable balance between predictive accuracy and robust class separability, supporting its reliability and suitability for multi-class brain tumor classification.

### 5.5 Statistical significance evaluation

To validate that the obtained results are not due to random chance, one-sample t-tests were conducted against a random baseline (accuracy = 0.333, AUC = 0.5). As shown in Table 8, all metrics achieve **p-values** < **0.001**, indicating high statistical significance.

Extremely large Cohen’s d values further confirm that the observed improvements are not only statistically significant but also practically meaningful.

**Table 8:** Statistical significance analysis on Dataset II.

Metric	Mean ± STD	T-stat	P-value	Cohen’s d
Accuracy	0.9318±0.0080	105.18	<0.001	60.73
AUC Score	0.9893±0.0020	349.72	<0.001	201.91
Macro F1-score	0.9260±0.0046	181.63	<0.001	104.86

### 5.6 State-of-the art comparison analysis

The comparative analysis in Table 9 depicts the advancement of the proposed ResSGANet over both traditional CNN-based methods and contemporary deep learning architectures. Traditional models such as ResNet50 (Accuracy = 90.39%), DenseNet121 (93.20%), and MobileNetV3 (92.15%) demonstrate moderate performance; however, they remain low in both precision and recall, suggesting limitations in capturing the complex variability present in brain MRIs. Advanced approaches such as ViT (Accuracy = 96.07%) and MobileViT-V2 (97.96%) demonstrate improved performance, while other comparative methods like L2-SA + VGG (96.20%), Deep CNN-SVM (97.10%), and DCGAN (95.60%) also surpass traditional CNNs but still show either lower precision, recall, or reduced AUC performance.

In contrast, the ResSGANet achieves the highest accuracy (98.21%) with consistent performance across all metrics: precision (97.91%), recall (97.87%), F1-score (97.87%), and an almost perfect AUC of 0.9986. These improvements highlight the effectiveness of combining a ResNet backbone with the Swin Transformer and dual attention strategies (gated + global), which significantly enhance feature extraction and class discrimination. ResSGANet not only outperforms traditional CNNs and transformer-based baselines but also maintains superior results across all metrics, underscoring its robustness and reliability. This makes ResSGANet a strong candidate for clinical applications where accurate and dependable brain tumor classification is critical.

The fold-wise evaluation illustrated in Table 10 suggests that the proposed model shows consistent improvements over baseline models, with significantly higher accuracy across all folds. Standard CNNs, such as AlexNet (Avg = 89.88%), VGG-19 (92.37%), and EffNetB0 (92.87%), achieved the lowest accuracies, highlighting their difficulty in generalizing complex tumor morphology. More competitive networks, including ResNet-50 (95.40%), Xception (95.16%), and SqueezeNet (94.05%), performed better due to deeper or more efficient feature representations, yet their accuracy remained unstable across folds. ARM-Net provided greater stability and accuracy with an average of 96.65%, showing strong results across folds, particularly 96.67% in Fold-3.

**Table 9:** Comparison of proposed ResSGANet with existing methods.

Method	Accuracy	Precision	Recall	F1 score	AUC
L2-SA + VGG ^52^	0.9620	0.9530	0.9570	0.9550	0.970
Soft Attention CNN ^53^	0.9510	0.9510	0.9510	0.9510	–
Deep CNN-SVM ^54^	0.9710	0.9730	0.9760	0.9750	–
DCGAN ^55^	0.9560	0.9530	0.9510	0.9500	0.970
ResNet50 ^56^	0.9039	0.9000	0.9005	0.9010	–
DenseNet121 ^57^	0.9320	0.9295	0.9275	0.9285	–
MobileNetV3 ^58^	0.9215	0.9128	0.9107	0.9124	–
ViT ^59^	0.9607	0.9577	0.9534	0.9551	–
MobileViT-V2 ^60^	0.9796	0.9767	0.9718	0.9754	–
Proposed ResSGANet	**0.9821**	**0.9791**	**0.9787**	**0.9787**	**0.9986**

In contrast, the ResSGA-Net demonstrated superior accuracy across all folds, with 97.91% (Fold-1), 97.89% (Fold-2), and 98.48% (Fold-3), resulting in an overall average of 98.09%. Importantly, accuracy remained consistently above 97.8% across folds, indicating strong generalizability and minimal sensitivity to data partitioning. This performance highlights the additive benefit of combining the ResNet backbone with the Swin Transformer and dual attention mechanisms, which collectively enhanced feature discriminating across tumor classes. Therefore, the proposed framework not only surpasses traditional CNN and transformer-based models but also establishes a new benchmark for brain MRI tumor classification.

**Table 10:** Fold-wise performance comparison of proposed model with baselines using dataset I.

Model	Fold-1	Fold-2	Fold-3	Avg (3 Folds)
VGG-16	94.10	91.61	94.93	93.55
VGG-19	93.35	90.42	93.35	92.37
SqueezeNet	95.20	91.75	95.20	94.05
Xception	96.12	93.22	96.15	95.16
ResNet-50	96.30	94.10	95.80	95.40
AlexNet	90.22	90.27	89.16	89.88
EffNetB0	93.54	92.43	92.65	92.87
ARM-Net	97.41	95.87	96.67	96.65
Proposed Model	**97.91**	**97.89**	**98.48**	**98.09**

### 5.7 Ablation study

The ablation study successfully evaluates in Table 12 the performance of the intended ReSGA-Net model that combines a ResNet50 with a Swin Transformer and individual Gated and Global Attention, against a version not using attention mechanisms consisting only of a ResNet50 + Swin Transformer. Of thirty possible comparisons through the models key metrics (accuracy, AUC score, loss), the ReSGA-Net (Proposed) model consistently outperformed the Without Attention (ResNet50 + Swin) model. In the ReSGA-Net (Proposed) model, the fold-wise results consistently demonstrated the power of the model, with accuracies of 0.9808 Fold-1, 0.9795 Fold-2, 0.9859 Fold-3, and an average accuracy result of 0.9820. The AUC score also remained strong with an average of 0.9986, indicating the high potential for distinguishing between tumor and non-tumor cases. The loss average was relatively low at an average of 0.1171, indicating an efficient training, learning, and adaption, while not demonstrating overfitting.

Conversely, when attention is removed completely in the Without Attention (ResNet50 + Swin) approach, the model experiences a reduction in performance with an accurate performance of 0.9802 and fold-wise accuracies of 0.9801, 0.9780 and 0.9825. Although still high, the AUC score of 0.9975 was lower than that of the proposed model performance, so it seems that a manner to separate features between classes, AUC-wise, lies in the attention mechanisms. The model Representation Loss increased slightly as well (average loss of 0.1198, versus the 0.1171 average loss of the proposed model).

In conclusion, the ablation study elucidated the value of the Gated and Global Attention mechanisms in improving the accuracy and stability of the model. Removing Gated and Global Attention mechanisms from the proposed model brought about a small, but noticeable decrease in performance, suggesting that those attention modules help to refine feature extraction, and help the model differentiate a greater range of complex patterns whilst performing brain tumor identification.

To address concerns regarding the extent of the preprocessing and augmentation pipeline, an ablation study was conducted on Dataset II to isolate the individual contribution of different augmentation groups.

The ablation results further substantiate that the performance gains of ResSGA-Net do not arise from simple architectural stacking. Removing either the attention module or the Swin Transformer leads to a consistent and statistically significant degradation in accuracy, macro F1-score, and AUC. Importantly, the magnitude of degradation differs depending on which component is removed, indicating non-redundant functional contributions.

**Table 11:** Ablation study of data augmentation strategies on Dataset II.

Augmentation setting	Accuracy	AUC	Macro F1	validation loss
A0: No Augmentation	0.901	0.972	0.898	0.312
A1: Intensity-based Only	0.917	0.981	0.914	0.289
A2: + Geometric (Rotation/Flip)	0.928	0.986	0.923	0.271
A3: Full Pipeline (Proposed)	**0.932**	**0.989**	**0.926**	**0.261**

Specifically, removing attention mechanisms primarily affects class-wise recall and increases confusion between glioma and pituitary tumors, while removing the transformer reduces global discriminative capacity as reflected by lower AUC values. These complementary failure modes confirm that each component addresses a distinct modeling challenge, validating the necessity of the proposed integration strategy. Table 11 summarizes the results.

Without any augmentation (A0), the model achieves reasonable performance; however, accuracy and AUC are notably lower, indicating limited generalization. Introducing clinically realistic intensity-based augmentations (A1), such as brightness and contrast variations, leads to consistent performance improvements, suggesting enhanced robustness to scanner-dependent intensity variations.

Adding moderate geometric augmentations (A2), including small rotations and horizontal flipping, further improves accuracy and macro F1-score. These transformations preserve anatomical consistency and reflect plausible variations in patient positioning during MRI acquisition.

The full augmentation pipeline (A3) achieves the best overall performance; however, the observed gains are incremental rather than abrupt. Importantly, the improvement saturates, indicating that the model does not rely on artificial data diversity but instead benefits from improved generalization. The consistently high AUC values across all augmentation settings confirm that learned features are tumor-specific rather than augmentation-driven.

Overall, this analysis demonstrates that the proposed augmentation strategy enhances robustness while preserving clinically meaningful image characteristics, alleviating concerns regarding unrealistic feature learning.

Table 13 provides a comparison of the ReSGA-Net model that incorporates Gated and Global Attention with the Without Attention version that contains only ResNet50 + Swin Transformer for all experimentation over the four tumor classes, Meningioma, Glioma, Pituitary, and No Tumor, and for each fold in terms of precision, recall, and F1-score.

**Table 12:** Ablation study: Performance of ReSGA-Net with and without attention mechanisms.

Model variant	Fold	Accuracy	AUC score	Loss	Epochs
ReSGA-Net (Proposed)	1	0.9808	0.9988	0.1038	50
	2	0.9795	0.9982	0.1489	50
	3	0.9859	0.9987	0.0986	50
Average	–	**0.9820**	**0.9986**	**0.1171**	–

Without Attention (ResNet50 + Swin)	1	0.9801	0.9970	0.1069	50
	2	0.9780	0.9975	0.1539	50
	3	0.9825	0.9981	0.0986	50
Average	–	**0.9802**	**0.9975**	**0.1198**	–

For Meningioma, ReSGA-Net displays a strong and consistent performance over all folds, with high precision and recall values. In Fold-1 the precision is 0.9437 and recall is 0.9991 with an F1-score of 0.9718. The Without Attention version performs similarly with slight variations in precision from 0.9421–0.9707, recall from 0.9801–0.9980, but slightly lower F1-scores from 0.9707–0.9734. This indicates that the attention mechanisms provide an improved overall balance between precision and recall, giving ReSGA-Net a slight advantage for identifying Meningioma tumors.

For Pituitary, ReSGA-Net obtained perfect precision (1.0000) in Fold-1, with Recall lower (0.9524) result and an F1-score of 0.9756. The Without Attention model also achieved high precision (1.00), but testing for Recall performed a little worse, especially in Fold-2 (0.9301), caused a lower F1-score (0.9616–0.9687). This performance difference in F1-score suggests the attention mechanisms are assisting in improved recall for Pituitary class, aiding in correctly classifying the cases of tumors in the images.

Lastly, considering the No Tumor class both models have performed excellently, with ReSGA-Net providing near perfect F1-scores (0.9914–0.9993) across both case folds. The Without Attention model also utilized demonstrates high F1-scores (0.9908–0.9975) but attention yielded a slight advantage across both precision and Recall for the ReSGA-Net model, thus, suggesting that the attention mechanisms in the model provide a more robust classification for healthy cases and crediting for their effective role in the model improvements.

In summary, ReSGA-Net’s Gated and Global Attention methods, clearly surpass the baseline Without Attention model, particularly in tumor classes (i.e., glioma and pituitary). The attention layers assist with feature extraction and class classification accuracy, thus improving model performance in glioma and pituitary. For the no tumor class, there is a positive classification outcome in both models, however, the consideration of overall precision and recall, reveals that ReSGA-Net performance mediates with respect to the baseline without attention model. It becomes apparent that the attention-based model is learning and integrating the task of identifying visual characteristics in all of the tumor classes tested.

**Table 13:** Class-wise and fold-wise comparison of ReSGA-Net and without attention (ResNet50 + Swin).

Tumor class	Fold	ReSGA-Net			Without attention		
		Precision	Recall	F1	Precision	Recall	F1
Meningioma	1	0.9437	0.9991	0.9718	0.9421	0.9980	0.9707
	2	0.9478	0.9892	0.9663	0.9463	0.9882	0.9646
	3	0.9721	0.9814	0.9767	0.9707	0.9801	0.9734

Glioma	1	0.9812	0.9819	0.9815	0.9801	0.9804	0.9802
	2	0.9745	0.9923	0.9833	0.9731	0.9904	0.9811
	3	0.9767	0.9991	0.9878	0.9743	0.9983	0.9863

Pituitary	1	1.0000	0.9524	0.9756	1.0000	0.9512	0.9724
	2	0.9988	0.9321	0.9642	0.9981	0.9301	0.9616
	3	0.9913	0.9498	0.9701	0.9901	0.9467	0.9687

No Tumor	1	0.9917	0.9912	0.9914	0.9904	0.9906	0.9908
	2	0.9945	0.9991	0.9968	0.9934	0.9981	0.9945
	3	0.9992	0.9994	0.9993	0.9984	0.9987	0.9975

## 6 Discussion

The automated classification of brain tumors from MRI images remains a critical and challenging task due to tumor heterogeneity, class imbalance, and subtle inter-class visual similarities. As highlighted in prior studies, conventional CNN-based models have demonstrated strong performance in extracting local spatial features but often struggle to capture global contextual dependencies and tumor morphology variations.^11^^,^^12^ These limitations can lead to misclassification, particularly when tumor types exhibit overlapping intensity patterns or similar anatomical structures. Recent advances incorporating attention mechanisms have shown promise in mitigating these issues by allowing models to focus on tumor-relevant regions while suppressing background noise. Channel-wise and spatial attention strategies have been reported to significantly improve discriminative feature learning in brain tumor classification tasks.^20^^,^^30^ However, attention-enhanced CNNs remain constrained by their localized receptive fields, which may limit their ability to model long-range dependencies inherent in complex tumor structures.

Transformer-based architectures, particularly Swin Transformer variants, have recently emerged as powerful alternatives due to their hierarchical self-attention mechanisms capable of capturing both local and global contextual information.^17^^,^^48^ These models have demonstrated superior generalization and robustness, especially when combined with transfer learning and advanced augmentation strategies. Nevertheless, applying transformers directly to raw convolutional features can introduce sensitivity to noise and increase computational complexity, as noted in prior works.^18^^,^^46^ The proposed ResSGA-Net is designed to address these limitations through a structured and task-driven integration of convolutional, attention-based, and transformer-based components. Unlike parallel or early-fusion hybrid architectures reported in the literature, ResSGA-Net adopts a sequential refinement strategy. Global attention is first employed to model inter-regional dependencies across the entire MRI slice, followed by gated attention to selectively amplify tumor-relevant activations. This attention-refined representation is then processed by a Swin Transformer, enabling global contextual reasoning on semantically enriched features rather than raw convolutional outputs.

The effectiveness of this design is empirically validated by the comprehensive evaluation on Dataset II. The model achieved a macro-averaged AUC of 0.989 and consistently high F1-scores across tumor classes, outperforming many existing CNN- and attention-based approaches reported in recent studies.^13^^,^^16^ Fold-wise analysis further demonstrated stable generalization, with minimal performance variance across cross-validation splits. Confusion matrix analysis revealed that most misclassifications occurred between glioma and pituitary tumors, which aligns with known clinical challenges due to morphological similarities.^2^ Importantly, meningioma cases were consistently classified with high precision and recall, indicating effective feature discrimination. The robustness of ResSGA-Net is further supported by training dynamics and ROC analysis. Smooth convergence of training and validation loss curves across folds indicates effective regularization and controlled learning behavior. Near-perfect ROC curves across all folds confirm strong class separability over varying decision thresholds, a crucial requirement for diagnostic imaging platforms where sensitivity and specificity trade-offs directly impact clinical decisions.

A key concern in automated MRI analysis is the reliance on extensive data augmentation, which may introduce unrealistic image variations. In this study, ablation analysis demonstrated that while augmentation contributes to improved generalization, performance gains saturate beyond clinically plausible transformations. This indicates that ResSGA-Net learns tumor-specific representations rather than relying on artificial data diversity, addressing concerns raised in previous studies regarding augmentation-driven bias.^49^^,^^50^ From a clinical perspective, the high confidence predictions, low performance variance, and strong statistical significance observed across all evaluation metrics suggest that ResSGA-Net has the potential to be integrated into diagnostic imaging workflows. Automated, non-invasive classification systems such as the proposed model can assist radiologists by reducing interpretation time, minimizing human error, and supporting early therapeutic intervention, as emphasized in recent neuro-oncological imaging studies.^5^^,^^7^

Overall, when positioned within the context of existing literature, ResSGA-Net advances current brain tumor classification research by establishing an effective and empirically validated integration strategy that balances accuracy, robustness, and computational feasibility. Rather than introducing isolated architectural novelties, the proposed framework demonstrates how carefully orchestrated interactions between residual learning, attention refinement, and transformer-based contextual modeling can yield clinically meaningful performance improvements.

## 7 Conclusion

This work presented ResSGA-Net, a hybrid deep learning framework for automated brain tumor classification from MRI images. By integrating a ResNet50 backbone with global and gated attention mechanisms and a Swin Transformer, the proposed architecture effectively captures multi-scale local features and long-range contextual information critical for distinguishing complex tumor patterns. Extensive experiments conducted on two benchmark datasets demonstrated that ResSGA-Net achieves state-of-the-art performance, with high accuracy, strong class separability, and consistent generalization across cross-validation folds. Statistical significance analysis confirmed that the improvements are not attributable to random chance, while ablation studies verified the individual contributions of attention mechanisms and data augmentation strategies. Importantly, augmentation analysis showed that performance gains stem from enhanced robustness rather than unrealistic image transformations, ensuring clinically meaningful feature learning.

Qualitative evaluations, including confusion matrices, training dynamics, ROC curves, and confidence-based visualizations, further validated the stability, reliability, and interpretability of the model’s predictions. These results collectively indicate that ResSGA-Net provides a robust and dependable solution for multi-class brain tumor classification. Future work will focus on extending the framework to multi-modal MRI sequences, incorporating uncertainty estimation and explainability mechanisms, and validating performance on larger, multi-institutional clinical datasets. Such extensions will further strengthen the applicability of ResSGA-Net as a decision-support tool in real-world neuro-oncological workflows.

## CRediT authorship contribution statement

**Yucheng Guan:** Writing – original draft. **Ahmad Alshammari:** Writing – review & editing, Methodology, Data curation. **Yu Wang:** Visualization, Validation, Methodology. **Jahan Zeb Gul:** Writing – review & editing, Software, Funding acquisition, Formal analysis. **Azhar Imran:** Writing – review & editing, Resources, Project administration.

## Declaration of competing interest

The authors declare that they have no known competing financial interests or personal relationships that could have appeared to influence the work reported in this paper.

## Footnotes

f https://www.kaggle.com/datasets/sartajbhuvaji/brain-tumor-classification-mri.

g https://www.kaggle.com/datasets/rahimanshu/figshare-brain-tumor-classification.
